# Relationship between blood Lead status and anemia in Ugandan children with malaria infection

**DOI:** 10.1186/s12887-020-02412-2

**Published:** 2020-11-14

**Authors:** Ambrose Mukisa, Denis Kasozi, Claire Aguttu, Peter C. Vuzi, Joseph Kyambadde

**Affiliations:** Department of Biochemistry and Sports Science, College of Natural Sciences, Makerere University, Kampala, Uganda

**Keywords:** Blood Lead, Malaria parasites, Anemia, Iron deficiency, Free erythrocyte protoporphyrins

## Abstract

**Background:**

In Uganda, childhood anemia remains a health challenge and is associated with malaria infection as well as iron deficiency. Iron deficiency is intertwined with nutritional status, age and other comorbidities including helminths and Lead toxicity. Environmental Lead levels accounts for one’s blood Lead (BL) levels. Blood Lead competitively blocks iron absorption, inhibits hemoglobin (Hb) biosynthesis and elevates free erythrocyte protoporphyrin (FEP) levels. Lead toxicity’s contribution towards anemia pathogenesis, especially during malaria infection has not been studied. Concomitant exposure to both malaria infection and Lead pollution, exacerbates the anemia status. This study therefore aimed at expounding the anemia status of these Ugandan children aged under 5years who are exposed to both malaria infection and environmental Lead pollution.

**Methods:**

Briefly, venous blood samples from 198 children were microscopically assayed for malaria parasite density (PD), and hemoglobin (Hb) concentrations using the cyanmethemoglobin method, while BL and FEP levels were determined by the standard atomic absorption spectrophotometric and fluorometric methods respectively.

**Results:**

One hundred and fifty-one (76.3%) of the children analyzed had moderate anemia (Hb <10>5 g/dL) with Means of BLL=8.6 µg/dL, Hb =7.5 g/dL, FEP/Hb =8.3 µg/g and PD =3.21×10^3^ parasites / µL, while eight (4%) were severely anemic (<5 g/dL). Regression analysis and statistical correlation between PD and Hb (r = -0.231, R^2^= 0.15 P-value < 0.001) was negative and weak as compared to that between FEP/Hb and Hb (r = -0.6, R^2^=0.572 P-value=0.001).

**Conclusion:**

Based on the study’s findings, we conclude that BL significantly contributes to the pathogenesis of anemia and therefore its co-existence with malaria infection in the host exacerbates the anemia status.

## Background

Like many malaria holoendemic developing countries, Uganda is faced with health-threatening diseases including anemia. Majority (60%) of the Uganda’s urban poor live in social disadvantaged slums like Katanga, Kampala [1, 2]. In such areas people live next to industries, workshops, motor garages, metal crafts yards, battery recycling plants, mosquito breeding grounds, and landfills predisposing them to frequent malaria infections as well as Lead intoxication. Several studies report elevated environmental Lead levels in water sources, soils, foodstuffs and air around Kampala city [3].

Malaria and Lead pollution geographically overlap, with water acting as sinks to Lead contaminated runoffs as well as bleeding ground for mosquitos. According to a study by [4], malaria infection accounts for up to 40% of all hospital outpatient visits, 25% of all hospital admissions, and 14% of all hospital deaths [4, 5] despite government efforts to curb its transmission [6]. Already fifty-three percent of children under the age of 5 years in Katanga area are reported to be anemic [6–9] this being attributed to high malaria prevalence.

Although, malaria infections remain the key cause of anemia in Uganda, other neglected yet significant confounders contributing to its pathogenesis include Lead poisoning, nutritional status, and helminths among others [6–9]. Lead exposure accounts for an individual’s blood Lead level (BLL) and is more evident in developing fetus where it directly affects the hematopoietic system. Ninety-nine percent of all the BL sink in the erythrocytes and 80% of it bind the δ-aminolevulinic acid dehydratase (δ-ALAD) enzyme. This enzyme is believed to catalyze the formation of porphobilinogen from δ-aminolevulinic acid (ALA) [10]. During Lead intoxication, BL inhibits activity(s) of ALAD and limit the transfer of iron from endosomes to the cytoplasm resulting into fragile cell membrane which in turn shorten the lifespan of the circulating erythrocytes [11–13].

Blood Lead further triggers a reduction in red blood cells (RBC) production by specifically inhibiting Ferrochelatase a mitochondrial enzyme that catalyzes the insertion of iron into protoporphyrin during heme formation. Aminolevulinic acid synthetase (ALAS) a mitochondrial enzyme that catalyzes the formation of aminolevulinic acid (ALA) from succinyl CoA and glycine is also affected by BL [13–16]. In a normal heme synthesis system, the rate of iron formation and utilization is well balanced, however, this equilibrium is disturbed with insufficiency of iron.

High malaria burden, overproduction of protoporphyrin, iron deficiency, inhibited ALAD and impaired ferro chelatase activity accounts for anemia pathogenesis. During malaria infection, intrinsic and extrinsic challenges are elevated and they induce iron deficiency anemia whose persistence elevates the rate at which Lead is absorbed [17–19]. In addition to other symptoms, malaria infection becomes fatal with severe anemia. Coupled with severe anemia, malaria is a result of massive erythrocytes lysis because of raising parasite density which in turn causes rupture of parasitized and non-parasitized red cells. Its persistence activates the splenic and other macrophages activities for phagocytosis [20] resulting into severe anemia.

Reduced cellular iron concentrations on the other hand enhances Lead’s effects on major synthesis reactions. For example, ferrochelatase enzyme which is specifically sensitive to low iron levels is affected by lead toxicity [21]. Also zinc instead of iron is incorporated into protoporphyrin (PPN) resulting in elevated levels of zinc protoporphyrin (ZPP) [22]. Zinc protoporphyrin or FEP concentrations are key biomarkers of heme synthesis status, and therefore, their elevation in concentration is associated with iron deficiency due to heme synthesis disorders [23, 24].

Since red cells are at the centre of anemia pathogenesis, as well as home to both the malaria parasites and BL, their co-existence propagates the host cell’s survival challenges. The severity of anemia is dependent on the host’s age and nutrition status [25, 26]. In addition, the combined aftermath of malaria parasites and BL among individuals living in malaria-endemic regions heighten the progression to severe anemia. This study therefore aimed at expounding the anemia status of these Ugandan children who are exposed to both malaria infection and environmental lead pollution. In this study, hemoglobin (Hb) concentration was used as a measure of one’s anemia status, and FEP/Hb ratio for heme synthesis status.

### Methods

This was a cross-sectional study on children aged 6 -60months living in Katanga area- Kampala city. The children were first screened for Plasmodium parasite infection before recruitment using rapid diagnostic kits. Children with malaria negative test, HIV positive test, blood transfusion history and signs of malnutrition were excluded from the study. Five (5) mls of venous blood from 198 malaria positive children were collected into EDTA by Qualified Nurses and Technicians. The samples were then transported on ice to Makerere University, Biochemistry Department Laboratory, stratified according to malaria parasite density and kept at 4^0^C awaiting various analyses and determinations.

### Determination of parasite density by thick smear method

Thick smears were prepared as described by [27], air dried, stained with 10% Leishman without fixing and examined under a CX 21 Olympus microscope. Five hundred leukocytes plus the number of malaria parasites seen in the same field were recorded. The number of malaria parasites per microliter (parasite/µL) of blood was expressed as the reciprocal of the mean counts in the three slides divided by the leukocyte counts, multiplied by a factor of 8000 i.e.

$$ \mathrm{Parasites}/\upmu \mathrm{L}\;\mathrm{blood}=\frac{\mathrm{Number}\kern0.17em \mathrm{of}\kern0.17em \mathrm{parasites}\kern0.17em \mathrm{counted}\times 8000\;\mathrm{white}\kern0.17em \mathrm{blood}\kern0.17em \mathrm{cells}/\upmu \mathrm{L}}{\mathrm{No}.\mathrm{of}\kern0.17em \mathrm{white}\kern0.17em \mathrm{blood}\kern0.17em \mathrm{cells}\kern0.17em \mathrm{counted}} $$

### Determination of blood Lead levels by atomic absorption spectrometry

Blood Lead levels were determined following a method described by [28] using an atomic absorption spectrophotometer (Agilent 2000 series) equipped with a graphite tube atomizer and deuterium background correction facility. A hollow-cathode Lead lamp with a working current of 5 mA, 283.3 nm spectral line and 0.5 nm bandwidth was used. Aliquots (500 µl) of whole blood melted with 1.2 ml of 0.5% Triton X-100 and 1% (NH_4_)_2_HPO_4_ solution were added to 1.8 ml of deionized water and 1.5 ml of 20% Trichloroacetic acid (TCA) and vortex mixed. The samples were centrifuged for 20 min at 5000 rpm and 10 µl of the supernatant injected onto the graphite tube. The calibration curve was drawn using a standard addition method as described in M-572 of INSPQ’s Toxicology Laboratory method.

### Fluorospectrophotometric quantification of free erythrocyte protoporphyrins

The FEP was measured following a method described by [29] using a fluorospectrophotometer set at 405 nm excitation 610 nm emissions. The porphyrins were extracted by adding 20 µl aliquots of whole blood to a solution containing 100 µL of 10% ammonium sulfate and 5% celite and vortex mixed for 10sec. 400 µl of 95% ethanol was then added and vortex mixed for more 20 seconds. This was followed by addition of 600 µl of acetone and further vortex mixing for 20 seconds. All the samples were put on an ice bath for 20 min, vortex mixed for 20 sec. and centrifuged at 4 °C for 10 min. After which the supernatants were harvested into small borosilicate tubes and aliquots of 300 µl mixed with 300 µl of a solution containing propylene glycol and 1.5 N HCI, (4:1), let to stand for 20 min before reading at 405 nm excitation and 610 emissions. The FEP blood concentration was calculated using the following formulae;

$$ \mathrm{FEP}\ \left(\upmu \mathrm{g}/\mathrm{dL}\ \mathrm{Blood}\right)=\mathrm{FEP}\ \upmu \mathrm{g}/100\ \mathrm{ml}\ \mathrm{extract}=\frac{{\mathrm{F}}_{\mathrm{z}}\times {\mathrm{C}}_{\mathrm{s}}\times 2.7\times 100}{{\mathrm{F}}_{\mathrm{s}}\times 1.1\times 0.2} $$

Where Fz is the sample fluorescence, Cs concentration of the standard, Fs is the fluorescence of the standard, 2.7 is the final volume of HCl phase, 100 is the conversion factor to 100 ml of extract, 1.1 conversion factor for protoporphyrin measured against a coproporphyrin standard and 0.2 (20 µl) is the original blood volume measured. The FEP/Hb ratio expressed as microgram Hb per gram Hb was calculated by dividing the FEP/dL RBC by gram Hb/dL.

### Colorimetric determination of hemoglobin levels by blood cyanmethemoglobin reaction

Hemoglobin levels were determined by cyanmethemoglobin reaction method [30]. Aliquots 100 µl of samples were made to a total volume of 1000 µl with reaction solution containing 200 mg of hexacyanoferrate III, 50 mg of potassium cyanide, 140 mg of potassium hydrogen phosphate and 1 ml of Triton X-100 in a liter of distilled water. Then incubated for 15 min at room temperature before reading at 540 nm, with the blank being the reaction reagent. Then 500 µl of standard hemoglobin standard (0.7 mg/ml) was diluted with 500 µl of the same reagent, treated as above and readings taken. The Hb concentration in g/dl was calculated using formula;

$$ \mathrm{Hb}\;\mathrm{concentration}\;\left(\mathrm{g}/\mathrm{dL}\right)=\frac{\mathrm{OD}\;\mathrm{sample}\times \mathrm{concentration}\kern0.17em \mathrm{of}\kern0.17em \mathrm{the}\kern0.17em \mathrm{standard}\left(\mathrm{mg}/\mathrm{dL}\right)}{\mathrm{OD}\;\mathrm{standard}\kern0.17em \mathrm{sample}} $$

Where OD = optical density or absorbance at 540 nm.

## Results

Of the 198 children enrolled in the study, 8/198 (4%) were severely anemic with < 5 g/dL, mean parasite density = 7.4 × 10^3^ parasites/ µL, mean BLL = 9.2 ± 4.3 µg/dL, an FEP/Hb = 7.4 ± 2.9 µg/g. Thirty nine (19.7%) of the study population were not anemic with a mean value of Hb > 10 g Hb/dL (WHO cut off reference), mean parasite density = 1.7 × 10^3^ parasites/ µL, mean BLL < 2 µg/dL, Mean FEP/Hb = 7.4 ± 2.9 µg /g. The details of the distribution are shown in Table 1 below

**Table 1:** Distribution of parasite density, BLL, FEP/Hb and hemoglobin levels among 198 study participants

No. of samples, N	Parasite density/ µL (× 10^3^)		Mean BLL (µg/dL)	Mean FEP/Hb (µg /g)	Mean Hb (g/ dL)
16	0 .1–1.1		10.3 ± 1.9	7.9 ± 3	7.0 ± 3.2
39	1.2–2.2		< 2	0.6	10.4 ± 2.7
42	2.3–3.3		10.3 ± 1.7	2.9 ± 3.2	8.5 ± 3.1
35	3.4–4.4		7.9 ± 2.1	3.9 ± 2.8	9.2 ± 3.0
34	4.5–5.5		7.9 ± 1.8	8.4 ± 3.2	9.3 ± 4.1
23	5.6–6.6		6.4 ± 1.8	4.9 ± 2.7	6.8 ± 3.3
8	6.7–7.7		9.7 ± 5.2	7.4 ± 2.0	4.9 ± 2.9
1	7.8–8.8		5.1	5.5	6.7
N = 198	Mean = 1.7 × 10^3^		Mean = 8.6	Mean = 4.4	Mean = 8.7

*Hb *Hemoglobin, *BLL *Blood Lead levels, *FEP *Free erythrocyte protoporphyrin

## Discussion

Several cellular biomarkers are used to measure the extent and effects of blood Lead (BL) on various biochemical systems. This study explored the levels of Free erythrocyte porphyrins (FEP) as a biomarker of heme biosynthesis disorder among the study participants. The heme biosynthesis process is inactivated by either iron deficiency, failed iron regulatory system or inhibited δ-aminolevulinic acid dehydratase (δ-ALAD) enzyme [31].

Free erythrocyte porphyrin levels of ˃50 µg/ dL are important indicator of heme biosynthesis disorder. Common causes of this disorder include iron deficiency due to malnutrition and poor iron absorption, elevated hepcidin levels, and ALAD inhibition by blood Lead [16–18, 20, 22, 29]. Findings of this study (Table 1) indicate that 88.2% (n = 175) of all study participants were moderately anemic Hb ˃5 g/dL < 10 g/dL. Of the 175 participants, 111 (56.6%) had normal functioning biosynthesis system (FEP levels ˃50 µg/ dL), although with moderate anemia. Therefore, the observed low levels of Hb, cannot be associated with iron deficiency. The candid cofounding factors for the observed low Hb levels therefore are oxidative stress and accumulated erythrocytic pyrimidine nucleotides. Oxidative may be caused by both BL and parasite density while the accumulated erythrocytic pyrimidine nucleotides are caused by inhibition of pyrimidine 5 nucleotidase enzyme (P5N) by BL. Enhanced oxidative challenges brought about by both parasites and Lead ions induces eryptosis, while the accumulated nucleotides cause cellular hemolysis. The study on oxidative challenges and pyrimidine nucleotide levels during both malaria infection and Lead toxicity was outside the scope of this work.

Considering the group of 39 participants (Table 1) with parasite density (PD) (1.2–2.2 × 10^3^ parasites/µL of blood), and no detectable BLL, there was a perfectly functioning heme biosynthesis system (FEP <10µg/ dL), with no anemia (mean Hb=10.4 g/ dL). It is likely that low PD by its own may not induce anemia especially to people living in malaria endemic areas. However, coexistence with another confounding factor like BL heightens the anemia pathogenesis. We can therefore hypothesize that progression to a severe anemia status is multifactorial and is exacerbated by having both malaria infection and blood Lead simultaneously.

Table 1, further shows a 4% (n = 8) severe anemic group of participants (Hb < 5 g/dL), with a fairly functioning heme synthesis system (FEP = 36.2 µg/ dL) still under 50 µg/ dL but with elevated PD. This gives an indication that the rate at which red blood cells (RBCs) were being destroyed by parasites surpassed their rate of synthesis hence the observed severe anemia status. Indiscriminate destruction of RBCs cause imbalances in the mediators of inflammation, Interleukin 6 cytokines (IL6) levels and this may affect the hepcidin expression [32, 33]. Interferon (IFN)-γ) known to induce the production of TRAIL (TNF-related apoptosis-inducing ligand) is also activated by elevated PD [34]. Again, the reported BLL (9.7 µg/ dL) of this group was high enough to induce oxidative stress. As well as inhibition of P5N enzyme hence eryptosis and hemolysis respectively, likely associated with the observed low Hb status. It is further observed (Table 1) that 16/198 participants that had low PD and elevated BLL were anemic (Hb = 7.0 g/dL) and suffered from iron deficiency anemia as indicated by the heme biosynthesis disorder (FEP ˃50 µg/ dL). The group’s low mean Hb levels could be attributed to elevated PD, BLL and other cofounding factors like iron deficiency due to malnutrition, inhibited iron absorption and inhibited (δ-ALAD) enzyme activity.

This study reports a lower mean BLL (= 8.6 µg/dL) compared to literature [35–37]. Furthermore, an uneven distribution of BLL among the study participants was observed. This is attributed to the fact that BL binds the RBC’s enzyme δ-ALAD which is polymorphic with two major alleles; δ-ALAD-1 and δ-ALAD-2 that differ in electronegativity [38]. Having high frequency of δ-ALAD-1 translates into higher BL burden than those with δ-ALAD-2 [39].

**Fig. 1:**
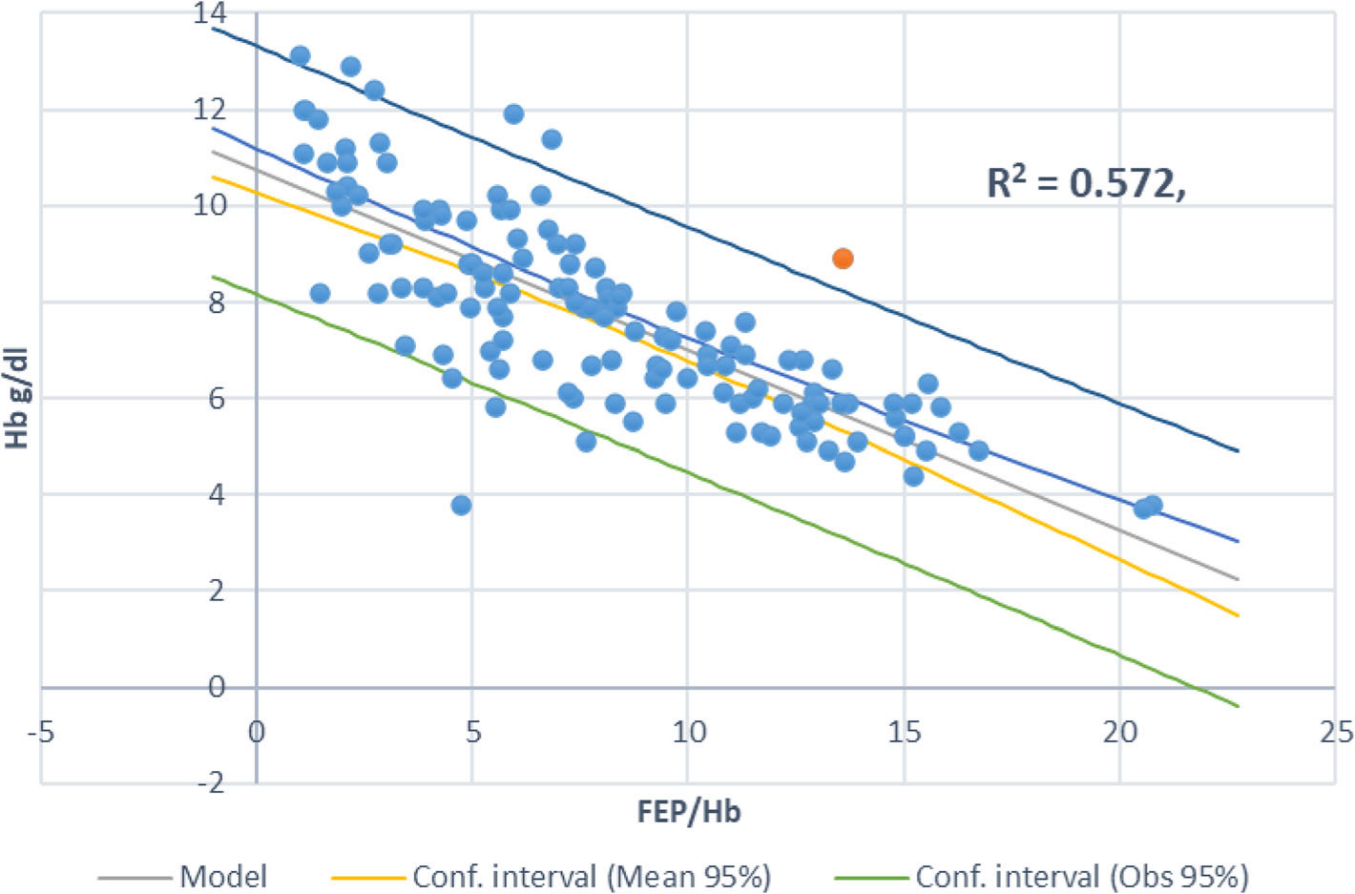
Scatter graph showing how FEP/Hb levels relate with hemoglobin levels of malaria infected children

**Fig. 2:**
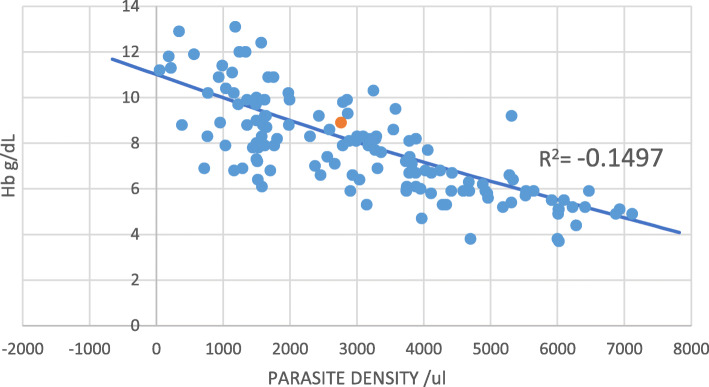
Scatter graph showing relationship between parasite density and hemoglobin levels among malaria infected children

A moderate interaction between the three variables (FEP/Hb, Parasite density and hemoglobin levels) after a multivariate analysis (Table 2) is reported. In addition, parasite density and BLL significantly correlated (r = 0.377, P = ˂0.001) to affect the anemia status of the host Table 2. Regression analysis (Pearson’s) models show that FEP: Hb µg /g ratio increases exponentially with the blood Pb while Hb decreased with increasing FEP (Fig. 1). Again, a negative and strong Pearson correlation between FEP: Hb µg /g and Hb levels (Table 2; Fig. 1) as compared to that of parasite density and hemoglobin levels (Fig. 2) were observed. This seem to concur with the argument that the etiology of severe anemia is multifactorial and therefore, there is need to study each of these contributing factors.

**Table 2:** Correlation coefficients r and p-values of different interacting variables

**Interacting variables**	**Correlation values r**	*p-values*
Parasite density and Hb	-0.231	0.035
Hb and BLL	0.552	<0.001
Hb and FEP/Hb	-0.572	<0.001
BLL and Parasite density	0.124	0.082
FEP/Hb, Parasite density Hb	0.377	<0.001

It can therefore be speculated that progression to severe anemia during malaria infection and Lead toxicity involves destruction of erythrocytes, inhibition of heme synthesis inhibition (ineffective erythropoiesis) and interference of hepcidin iron regulatory system. The increased erythrocytic clearance due to extrinsic and intrinsic challenges increase the susceptibility to phagocytosis and hence anemia. Reduced serum iron (substrate) availability further complicates the heme synthesis mechanism by disturbing the enzyme /substrate enzymatic reaction equation.

**Fig. 3:**
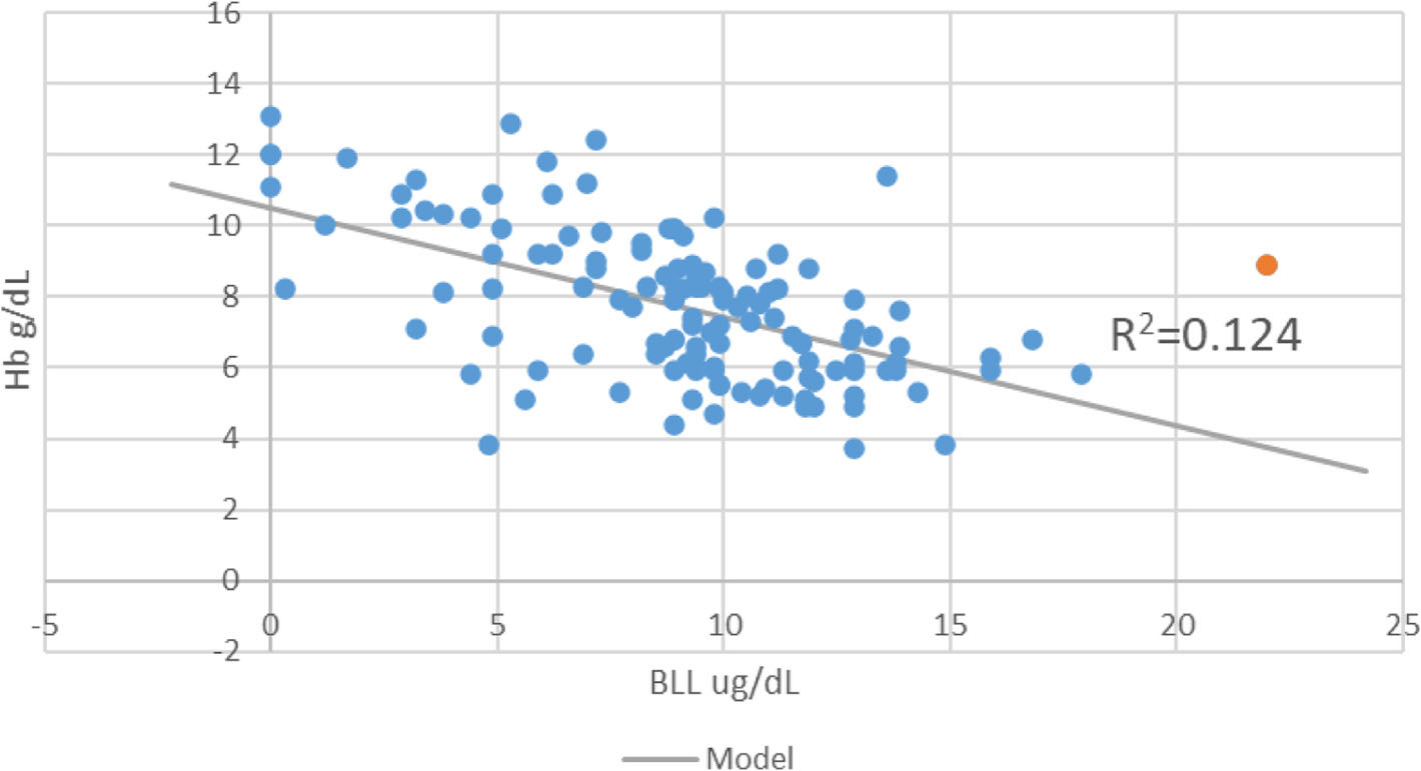
Scatter graph showing relationship between Blood Lead levels and hemoglobin levels among malaria infected children

However, this study did not find a direct relationship between high blood Lead concentrations with parasite density as previously reported. It is likely that as BLL increases, hepcidin expression is upregulated decreasing the available serum iron important for parasite survival. Blood Lead levels and malaria parasites (Table 2; Fig. 3) in the study population seems to support the argument that BLL had little or no direct effect on parasite density levels.

Children who participated in the study had no co-morbid diseases like HIV, sickle cell traits which are known confounding factors of anemia pathogenesis. This study therefore represents the first reported significant association between malaria and Lead poisoning during the anemia pathogenesis among the pediatric population in Uganda.

### Study limitations

This study did not assess the nutritional status of the participants which is an important confounding factor. We therefore recommend that future studies incorporate nutritional status of participants for a better understanding of the relationship between blood Lead levels and anemia during malaria infection.

## Conclusions

Based on this study findings, low blood Lead is a key confounding factor of anemia pathogenesis especially in children infected with malaria. Since both blood Lead and plasmodium infection geographically overlap with similar hematological consequences, their co–existence heightens the anemia status of the host.

We recommend a detailed study involving a bigger sample size to properly understand the effects and anemia pathogenesis during Lead exposure and Plasmodium malaria infection over a long period of time.

## Data Availability

The datasets used and/or analyzed during the current study are available from the corresponding author on reasonable request.

## Abbreviations

ALA: Aminolevulinic acid.
ALAD: Aminolevulinic acid dehydrogenase.
BL: Blood Lead.
BLL: Blood Lead levels.
FEP: Free erythrocyte protoporphyrin.
PD: Parasite density.
PPN: Protoporphyrin.
ZPP: Zinc protoporphyrin.
